# Global network analysis of drug tolerance, mode of action and virulence in methicillin-resistant *S. aureus*

**DOI:** 10.1186/1752-0509-5-68

**Published:** 2011-05-12

**Authors:** Ian M Overton, Shirley Graham, Katherine A Gould, Jason Hinds, Catherine H Botting, Sally Shirran, Geoffrey J Barton, Peter J Coote

**Affiliations:** 1 Biomedical Systems Analysis, MRC Human Genetics Unit, Institute of Genetics and Molecular Medicine, Western General Hospital, Crewe Road, Edinburgh EH4 2XU, UK; 2 School of Life Sciences Research, University of Dundee, Dow Street, Dundee DD1 5EH, UK; 3 Centre for Biomolecular Sciences, School of Biology, University of St Andrews, The North Haugh, St Andrews KY16 9ST, UK; 4 Bacterial Microarray Group, Division of Cellular and Molecular Medicine, St. George's, University of London, London SW17 0RE, UK

## Abstract

**Background:**

*Staphylococcus aureus *is a major human pathogen and strains resistant to existing treatments continue to emerge. Development of novel treatments is therefore important. Antimicrobial peptides represent a source of potential novel antibiotics to combat resistant bacteria such as Methicillin-Resistant *Staphylococcus aureus *(MRSA). A promising antimicrobial peptide is ranalexin, which has potent activity against Gram-positive bacteria, and particularly *S. aureus*. Understanding mode of action is a key component of drug discovery and network biology approaches enable a global, integrated view of microbial physiology, including mechanisms of antibiotic killing. We developed a systems-wide functional association network approach to integrate proteome and transcriptome profiles, enabling study of drug resistance and mode of action.

**Results:**

The functional association network was constructed by Bayesian logistic regression, providing a framework for identification of antimicrobial peptide (ranalexin) response modules from *S. aureus *MRSA-252 transcriptome and proteome profiling. These signatures of ranalexin treatment revealed multiple killing mechanisms, including cell wall activity. Cell wall effects were supported by gene disruption and osmotic fragility experiments. Furthermore, twenty-two novel virulence factors were inferred, while the VraRS two-component system and PhoU-mediated persister formation were implicated in MRSA tolerance to cationic antimicrobial peptides.

**Conclusions:**

This work demonstrates a powerful integrative approach to study drug resistance and mode of action. Our findings are informative to the development of novel therapeutic strategies against *Staphylococcus aureus *and particularly MRSA.

## Background

Methicillin Resistant *Staphylococcus aureus *(MRSA) is a major cause of morbidity and mortality [1-4]. Indeed, rates of MRSA infections have risen significantly in recent years [3,5]. Strains that are resistant to existing treatments continue to emerge and community-associated MRSA is a major global problem [3,4,6-10]. Therefore, the development of novel prevention and treatment strategies is a pressing concern.

Antimicrobial peptides (AMPs) are a potential source of novel antibiotics that may be developed to combat resistant bacteria such as MRSA [11]. AMPs are produced by virtually all living creatures as part of their innate defences and more than 880 have been described [12]. Ranalexin is a cationic 20 amino acid peptide, first isolated from the American bullfrog, *Rana catesbeiana*, and has a single intramolecular disulphide bond to form a heptapeptide ring at the carboxyl terminus [13]. Ranalexin has potent activity against Gram-positive bacteria *in vitro*, particularly *Staphylococcus aureus *[14]. Therefore, ranalexin offers therapeutic potential against staphylococcal infections, including MRSA.

Understanding the molecular mechanisms of antimicrobial action is an important facet of developing new therapeutic strategies, particularly where drug resistance is a problem [15,16]. Transcriptome and proteome profiling offers a powerful approach for studying antimicrobial inhibitory action [17-19]. In principle, the mRNA and protein profiles generated in response to the imposition of antimicrobial stress reflect modulation of particular cellular functions, and provide a signature of the type of stress imposed. For example, expression profiling has been applied to predict mode of action [17] and to identify molecular targets of uncharacterised antibiotics [18]. A network biology approach in combination with expression profiling enables systems analysis of drug mode of action, for example with networks of drug interactions [20] or gene regulation [21]; for a recent review see [22].

We took a systems-wide approach to integrate transcriptome and proteome profiling of drug-exposed bacteria with a high-confidence functional association network [23-25] that modelled pathway relationships for 95% of *S. aureus *MRSA-252 genes. MRSA-252 is an isolate of one of the most prevalent epidemic MRSA clones, EMRSA-16 [26]. This approach enabled inference of twenty-two novel MRSA virulence factors and novel complementary killing mechanisms for the antimicrobial peptide ranalexin, including effects at the cell wall. We also found evidence supporting involvement of the VraRS two-component system in cationic peptide resistance. Furthermore, FtsH was proposed as a candidate drug target, and a role was inferred for PhoU-mediated persister formation in *S. aureus *drug tolerance. These results demonstrate the power of this global functional association network approach to study drug resistance and mode of action.

## Results and discussion

### Ranalexin elicits significant changes in transcript and protein levels

**Figure 1:**
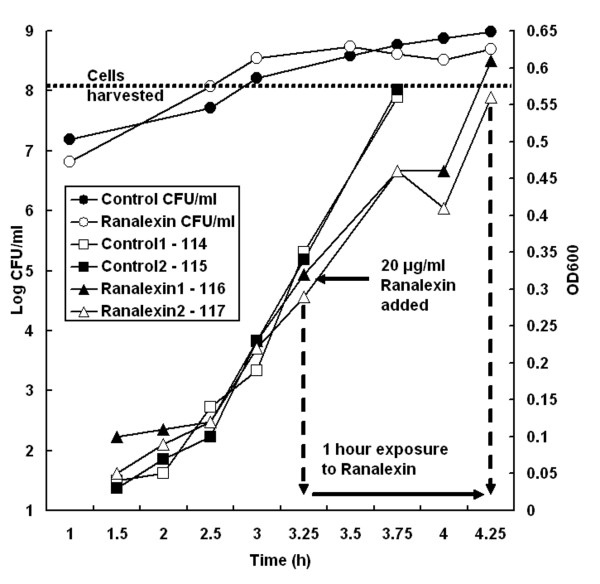
**Sublethal exposure to ranalexin (iTRAQ)**. Growth is shown for duplicate samples of MRSA-252 cultured with 20 μgml^-1 ^ranalexin (▲, Δ) and controls (■, □). Total CFU/ml is shown for ranalexin treated samples (○) and controls (●). Ranalexin treatment produces a temporary reduction of growth rate. Cells were harvested for proteome profiling by iTRAQ (Methods) at OD600 0.58, or one hour after ranalexin addition.

We determined a sublethal ranalexin concentration (20 μg/ml) that impaired, but did not abolish growth of MRSA-252 (Figure 1, Methods). Transcriptome and proteome profiling were applied to identify changes in the ranalexin exposed MRSA-252 cultures compared to controls (Methods). Microarrays identified 93 upregulated and 105 downregulated genes (>two-fold expression difference, *p *< 0.05), while iTRAQ LC-MS/MS coupled with ProQuant (Applied Biosystems) analysis identified 56 upregulated and 15 downregulated proteins by stringent criteria (Methods, Additional file 1 Tables S1-S3). No inconsistencies were observed between the transcriptome and proteome profiles. Overlap in Gene Ontology (GO) [27] annotation was observed for these datasets although there were only a few cases of direct overlap at the gene level. This is not uncommon for the integration of proteome and transcriptome data [28,29]. GO-based functional profiling of these transcripts and proteins identified 290 significantly enriched terms (*p *< 0.05, Methods, Additional file 1 Tables S4-S7), underlining the multi-faceted effects of ranalexin on MRSA.

### Global gene functional association network

A functional association network [23-25] was developed in order to give a probabilistic model of global gene function in MRSA-252, and to provide a framework for systems-wide analysis of the ranalexin response profiles. The UniProt [30] MRSA-252 genes were network nodes, while connections (edges) between genes (nodes) represented relationships in cell signalling and metabolism. Details of network construction and evaluation are given in Methods. Briefly, edges were generated using a Bayesian logistic regression approach, which integrated information from Gene Ontology [27] coannotations, STRING scores [31] and KEGG [32] pathways (Methods). The final network contained 2494 nodes (genes) and 19076 edges (connections), with a false positive rate (FPR) no greater than 3% over several independent test datasets (mean FPR 2.6%). Interestingly, these FPR values are similar to the estimated value of the functional association prior (4.7%), which suggests that a significant fraction of the 'false positives' may be genuine functional relationships that were not annotated in the test data. Overall, the network edges relate 94.5% of MRSA-252 genes with high confidence.

**Figure 2:**
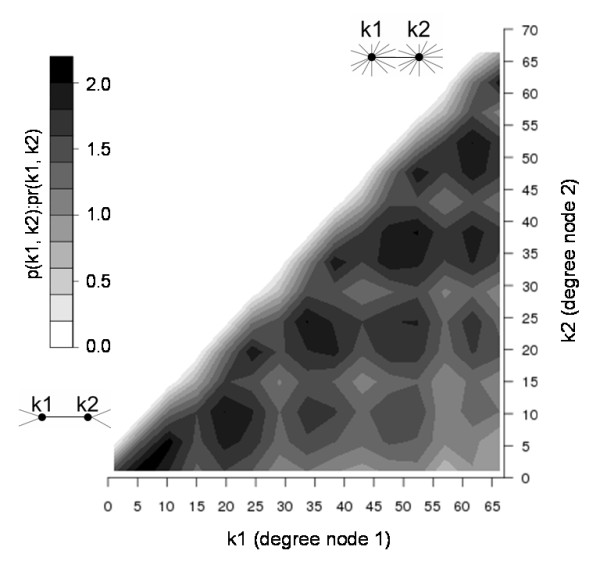
**Normalised probability of network degree pairs**. The axes represent the degree of interacting nodes (genes). The z axis scale indicates the ratio p(k1, k2)):pr(k1, k2). p(k1, k2) is the probability of observing an interacting pair of nodes in the MRSA network with degrees k1, k2 and pr(k1, k2) is same probability in the randomised MRSA network [102,103]. Therefore, the bottom left area of the plot represents connections between nodes that have low degree values, the area at the top right represents connections between nodes that have high degree values. Individual pair values of p(k1, k2)):pr(k1, k2) range from to 0.01 to 34.0. Values given on x and y axes represent bin lower bounds, a cutoff was applied at degree of 70 due to degree pair sparseness above this value. The observed degree pair distribution reflects hierarchical structure with embedded modularity [34]. This figure was generated using R [95].

The node pair degree connectivity (Figure 2), as well as the network degree and clustering coefficient distributions (Additional File 2 Figures S1, S2), denote hierarchical structure with embedded modularity that has been previously observed for metabolic networks [33,34]. Indeed, the gene functional association network topology is closer to that of metabolic networks, rather than protein interaction networks which are less modular [24,33,34]. This result seems intuitively reasonable because gene (node) interactions in functional association networks are expected to be shared amongst all the members of a functional grouping, such as a signalling pathway, which would contribute to modular topology. Therefore, the *S. aureus *MRSA-252 gene association network structure fits well with further analysis based on module decomposition. The network was clustered into 597 putative functional modules using the MCL algorithm [35]. The transcriptome and proteome profiles were mapped into these modules, and significance was assessed against background distributions generated by network re-sampling (Methods). A total of eleven modules were found to be enriched with genes that displayed significantly altered expression in MRSA-252 cultures exposed to ranalexin. Of the eleven modules, five were upregulated and six downregulated; these results are summarised in Table 1. A total of 58 nodes outside these modules were classified as intermodular hubs (Methods, Additional file 1 Table S8), which link to multiple highly interconnected subnetworks and are putatively important regulators of system behaviour [36].

**Table 1:** Ranalexin response modules

Module description	Ranalexin Altered Gene Set	Additional Genes In Module	False discovery rate adjusted p-value
+ Val/Leu/Ile metabolism	SAR2141, SAR2143, SAR2144, SAR2146, SAR2147, SAR2148 (6/7* genes)	SAR2297	<9.0E^-5^*

+ Na^+^/H^+ ^antiporters (*mnh *operons)	SAR0629-SAR0635, SAR0908 (8/17* genes)	SAR0909-SAR0915, SAR0627, SAR0628	<9.0E^-5^*

+ Persister formation, phosphate transport (*pstSCAB-phoU operon*)	SAR1400-SAR1402, SAR1398 (4/4 genes)	-	1.2E^-4^

+ Chaperones, stress response	SAR0938, SAR1657, SAR1658 (3/3 genes)	-	0.027

+ Osmoprotectant transport	SAR2536-SAR2538 (3/6 genes)	3 genes: SAR2535, SAR0775, SAR0776	0.046

- Virulence factors (ESAT-6 system)	SAR0279, SAR0281-0284, SAR0287, SAR0288 (7/12* genes)	SAR0285, SAR0286, SAR0289, SAR0290, SAR0291	<1.9E^-4^*

- High-affinity metal ion transport (Fe)	SAR0787-SAR0790 (4/5 genes)	SAR1011	0.0019

- Virulence factors (Colonization, immuno-modulation)	SAR0567, SAR0847, SAR2508 SAR2709 (4/16 genes)	SA0566, SAR0842, SAR1102, SAR1103, SAR1223, SAR1489, SAR1802, SAR1841, SAR2383, SAR2421, SAR2580, SAR2734	0.0062

- High-affinity metal ion transport (Fe, Zn, Mn, Mo)	SAR0274, SAR0643, SAR2594, SAR2452 (4/9 genes)	SAR1633, SAR1928, SAR2361, SAR2543, SAR2544	0.0062

- Virulence (inferred from network)	SAR0292-SAR0294 (3/3 genes)	-	0.025

- Cell division	SAR1177-SAR1179 (3/4 genes)	SAR1175	0.028

A prefix '+' in the first column indicates modules with upregulated genes, a prefix '-' downregulated genes. *No instances of these module coverage levels were observed in the appropriate random sampling. Therefore the background frequency was set to 1/100000 for estimation of p-values.

### Impact on virulence and inference of novel determinants

**Figure 3:**
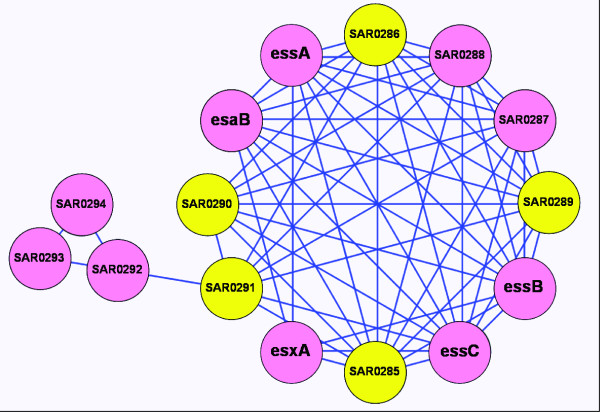
**ESAT-6 downregulated module and novel virulence factors**. Genes significantly downregulated by ranalexin (RanaDown) are shown in pink, other genes in yellow. Two significant network modules are shown (p ≤ 0.025, p ≤ 1.9E^-4^). Names (e.g. esxA) are given for genes that have been characterised, and these all represent ESAT-6 system components; otherwise locus identifiers are given (e.g. SAR0294). Network edges are shown as blue lines. Novel virulence roles were inferred for the SAR0292-0294 module and the seven uncharacterised genes in the ESAT-6 module (see main text). These network-based predictions are supported by several further lines of evidence, including predicted cellular localisation, domain matching and operon structure. This figure was generated in Cytoscape [104].

The genes significantly downregulated upon ranalexin exposure ('RanaDown') included all six of the MRSA-252 ESAT-6 secretion system components (Figure 3), which are central to *Staphylococcus aureus *pathogenesis [37]. A highly significant module (*p *≤ 1.9E^-4^, 7/12 nodes RanaDown) included five ESAT-6 components; the sixth MRSA-252 ESAT-6 gene (*esaA*, SAR0280) was not assigned to a module but was RanaDown and shared edges with 10/12 genes in the module. The two further RanaDown genes in this module (SAR0287, SAR0288) were relatively uncharacterised 'hypothetical' proteins [30,32]. Analysis of SAR0288 predicted six transmembrane regions [38] and found a match to the 'membrane ABC permease' domain PD089828 [39]; SAR0287 was predicted to be secreted or cell wall anchored [38], and matched to several protein families including conserved domains of unknown function (e.g. DUF1342), plus virulence-associated families (e.g. Sm_multidrug_ex, WXG100) [32,40]. These results agree with the network-inferred virulence function for SAR0287 and SAR0288. The remaining five genes in the module were also hypothetical, while all five matched to conserved domains of unknown function (e.g. DUF1415) and virulence-associated families (e.g. Endotoxin_N, WXG100) [32,40]. Consistent with roles in virulence for these remaining five genes, TMHMM2 [38] indicated two secreted/cell wall and one transmembrane protein, while predictions were not clear in two cases. The module had good correspondence with predicted operon structure [41,42], implying that the seven hypothetical genes may be co-regulated with the ESAT-6 system. The GO term 'pathogenesis' (GO:0009405) was significant for this module (*p *≤ 4.92E^-5^, 5/12 annotated genes, Table 2). Therefore, seven novel *S. aureus *(MRSA-252) virulence factors were inferred.

**Table 2:** Significant virulence modules.

Module Summary	Genes annotated with GO:0009405 ('pathogenesis')	Additional Genes In Module	False discovery rate adjusted p-value
Colonization, immunomodulation	SAR1102 SAR0842 SAR2580 SAR0567 SAR2383 SAR2709 SAR2508 SAR2734	SAR0566 SAR0847 SAR1841 SAR1103 **SAR1223 SAR2421 SAR1489 SAR1802**	6.64E^-8^

Staphylococcal enterotoxins	SAR1920 SAR1919 SAR1916 SAR1921 SAR1917 SAR1918	-	1.69E^-7^

Superantigen-like proteins	SAR0424 SAR0427 SAR0425 SAR0422 SAR0423	**SAR0426**	7.10E^-7^

Two-component (sensor, response regulator) systems, drug resistance	SAR0670 SAR2448 SAR1426 SAR2447 SAR1427 SAR0758 SAR0669	**SAR0068 **SAR1772 **SAR2167 **SAR1771 SAR1567 **SAR1332 SAR1331 SAR2449 **SAR1568 SAR0019 **SAR2450 SAR0760**	2.38E^-5^

ESAT-6 system	SAR0283SAR0282SAR0284SAR0279SAR0281	**SAR0285 SAR0287SAR0288 SAR0289 SAR0290 SAR0291 SAR0286**	4.92E^-5^

Superantigen-like proteins	SAR1141 SAR1140 SAR1139	-	6.36E^-5^

Superantigen-like proteins	SAR0431 SAR0428 SAR0429	-	6.36E^-5^

gamma-hemolysin	SAR2511 SAR2509 SAR2510	-	6.36E^-5^

Response regulators (MarR, SarR)	SAR2379 SAR0739 SAR2351	-	6.36E^-5^

Peptidases	SAR1022 SAR1020 SAR1021	SAR2716	2.22E^-4^

Modules with false discovery rate corrected Fisher p-values ≤ 0.05 and size >2 are given. All modules were manually inspected with reference to literature and relevant databases (e.g. [30,32]). Underlined locus identifiers were downregulated in response to ranalexin, none of the below loci were upregulated. Locus identifiers in bold are candidate novel virulence factors. Many genes that were not annotated with 'pathogenesis' in the Gene Ontology nevertheless had some association with virulence in the literature, and therefore are not indicated as candidate novel virulence factors in the table. Modules listed first 'Colonization, immunomodulation' and fifth 'ESAT-6 system' were also significant for the ranalexin downregulated gene set (Table 1).

Two significant RanaDown modules (*p *≤ 1.9E^-3^, 4/5 nodes RanaDown; *p *≤ 6.2E^-3^, 4/9 nodes RanaDown) were associated with high-affinity metal ion transport [32,40], which is crucial for establishment of infection [43,44]. The module with 4/5 RanaDown genes included SAR0787-SAR0790, representing the *sst *iron-uptake operon [45]. The fifth gene in this module (SAR1011) was a putative substrate binding protein for iron complex transport [32], which agrees with the functional relationship to the *sst *operon inferred from the network.

A further module (*p *≤ 6.2E^-3^, 4/16 nodes RanaDown) contained twelve genes with annotated virulence functions. These twelve genes were largely implicated in colonization (e.g. binding fibronectin, bone sialoproteins) and immuno-modulation (e.g. IgG binding, clumping factor A) [30,32,40,46]. The remaining four genes in this module encoded EbpS (SAR1489), a putative lyrA protease (SAR2421), a putative lytN cell wall hydrolase (SAR1223), and a conserved hypothetical protein (SAR1802) matching to the Pfam family 'hydrolase' [30,32,40]. Indeed, coordination of peptidoglycan hydrolase and virulence determinant expression has been observed in *S. aureus *[47,48]. All sixteen genes were known or predicted to encode cell wall anchored, transmembrane or secreted proteins [30,38,40]. The GO term 'pathogenesis' (GO:0009405) was significant for the module (*p *≤ 6.64E^-8^, 8/16 annotated genes). These data imply functions in virulence-coupled autolysis for SAR2421 (lyrA), SAR1223 (lytN) and SAR1802 (hypothetical). EbpS (SAR1489) is a transmembrane protein that binds (soluble) tropoelastin, and not associated with adhesion [49]. EbpS function is not well defined, however involvement in virulence is suggested by the network structure. A fully connected RanaDown module (*p *≤ 0.028, 3/3 nodes RanaDown) was composed of relatively uncharacterised, 'hypothetical' genes (SAR0292, SAR0293, SAR0294). Interestingly, the only edge from this module into the rest of the network connected with the ESAT-6 virulence module described above (Figure 3). Consistent with the network-based inference of virulence functions for these three genes, TMHMM2 indicated one transmembrane and two secreted/cell wall proteins, while database matches included conserved domains of unknown function (e.g. DUF600) and RTX toxin family (RTX) [38,40].

Additionally, the most significantly enriched GO term in the ranalexin downregulated gene set was 'pathogenesis' (GO:0009405, *p *≤ 7.78E^-9^, n = 14) (Additional file 1 Table S7). Many genes annotated with this GO term were strongly downregulated, for example: SAR2122 (*hld*, encoding delta-haemolysin; 0.19-fold); SAR1022 (*sspA*, encoding a glutamyl endopeptidase; 0.21-fold); SAR2648 (*ssaA1*, encoding staphylococcal secretory antigen precursor; 0.28-fold). Indeed, several *agr *locus genes additional to *hld *were RanaDown (*agrB *0.38-fold, *agrC *0.48-fold, *agrD *0.42-fold, *rnaIII *0.15-fold). Ranalexin dependent repression of virulence genes agrees with the upregulation of *sarA *attenuator SAR1374 (*msrR*, 2.24-fold), which inhibits *sarA*-dependent virulence gene expression [50]. Furthermore, two RanaDown genes (SAR0115, SAR2474) encoded putative HTH-type transcriptional regulators (*sarS, sarZ*) from the *sarA *family, that activate virulence factor transcription [51,52]. There is evidence linking traditional antibiotics to reduction of *S. aureus *virulence (reviewed in [53]), however this had not previously been reported for a cationic AMP. The observation that ranalexin results in repression of virulence factor expression has clear beneficial implications for potential clinical applications (e.g. catheter surfactant, nasal cream).

Prediction of additional *S. aureus *virulence factors was enabled by mapping GO annotations for 'pathogenesis' (GO:0009405) into the network (Methods). A total of ten modules were significant (*p *≤ 0.05, size >2), and were manually inspected (Table 2). These ten included the ESAT-6 and colonization-associated modules discussed above. Interestingly a significant module (*p *≤ 2.38E^-5 ^7/19 GO:0009405) had 15 known two-component sensors or regulators, and a further four poorly characterised genes which may form novel virulence-associated two-component systems. Manual inspection of these modules, with reference to literature and electronic resources (e.g. UniProt [30], KEGG [32]), indicated eight genes not previously annotated as virulence factors (SAR0068, SAR0426, SAR0760, SAR1331, SAR1332, SAR2167, SAR2449, SAR2450).

In summary, the above analysis indicated that ranalexin restricts *S. aureus *MRSA-252 pathogenicity, including the ESAT-6 system, and inferred twenty-two novel virulence factors.

### Ranalexin induces cell wall stress

The response to ranalexin included strong upregulation of several peptidoglycan synthesis genes, which suggests that ranalexin may act on the cell wall (Additional file 1 Table S4). Indeed, the Gene Ontology (GO) term 'peptidoglycan-based cell wall' (GO:0009274) was significantly enriched in genes upregulated by ranalexin (RanaUp) (*p *≤ 0.03, n = 3). The VraR (SAR1974) regulator protein of the vancomycin-resistance associated two-component system (VraSR) was RanaUp (2.45 fold). VraSR controls the expression of genes that are induced by vancomycin, many of which function in cell wall biosynthesis [54,55]. Several genes regulated by VraSR were found to be RanaUp, including SAR1461 (penicillin-binding, *pbp2*, 2.10-fold), SAR1964 (glycosyltransferase, *mgt*, 2.64-fold), SAR1030 (methicillin resistance-related, *fmt*, 2.45-fold) and SAR2442 (teicoplanin resistance, *tcaA*, 3.08-fold). The above genes include members of a general cell wall stimulon induced in response to cell wall active agents [56-58]. Moreover, VraSR is upregulated in vancomycin-intermediate strains of *S. aureus *and induced by exposure to cell wall active antibiotics such as glycopeptides and β-lactams [55,59]. A further RanaUp protein, FtsH (encoded by SAR0512, 1.23-fold), had the highest betweenness centrality in the network, degree of 74 (top 0.5%), and did not fall into a module. Therefore FtsH is an intermodular hub, which implies a key role in regulating system behaviour [36]. Indeed, FtsH is within the AAA (ATPases Associated with diverse cellular Activities) family and acts as a chaperone required for incorporation of penicillin binding proteins (PBPs) into the cell membrane [60,61]. As noted above, *pbp2 *was RanaUp and is part of a cell wall stimulon, while PBPs are upregulated in vancomycin-intermediate *S. aureus *(VISA) [57]. FtsH is also involved in response to osmotic stress and mutants are non-viable or have significantly attenuated virulence [61,62]. Considering the above, FtsH is proposed to be a key player in the MRSA response to antimicrobials such as ranalexin and a potential drug target. Indeed, these results suggest that a drug targeting FtsH would be particularly effective in combination with cell wall active agents. The upregulation of FtsH in concert with the cell wall stimulon further supports action of ranalexin at the cell wall. The largest ranalexin dependent induction of transcription was observed for SAR0584 (*vraX*, 23.89-fold), which is not well characterised. However, *vraX *was over-expressed in isolates of vancomycin-intermediate *S. aureus *(VISA) and showed >200-fold increased expression in vancomycin-sensitive *S. aureus *(VSSA) treated with vancomycin [59]. Overall, these data indicate that ranalexin caused cell wall stress similar to that produced by vancomycin - a cell wall active antibiotic.

RanaUp genes also included transcriptional regulatory proteins encoded by SAR1689 (GreA, 4.00-fold) and SAR0625 (SarA, 4.36-fold) that are known to be induced in the presence of cell wall active antibiotics [59,63]. A transcriptional attenuator of SarA, SAR1374 (*msrR*, 2.24-fold), was induced by ranalexin. Significantly, *msrR *is induced upon exposure to cell wall active antibiotics, while its deletion results in sensitivity to methicillin and teicoplanin [50]. An autolysis-deficient, teicoplanin-resistant strain of MRSA was found to upregulate *fmtA, sarA, tcaA, msrR, vraR *plus a large number of proteases [64]. As noted above, these genes were found to be RanaUp. Indeed, inactivation of MRSA methicillin resistance genes has been reported to increase susceptibility to the β-defensin and LL37 AMPs [65]. Evidence supporting upregulation of cell wall anabolism also included enrichment of the GO term 'aspartate family amino acid biosynthesis' in the RanaUp set (GO:0009067, *p *≤ 1.45E^-3^, n = 5). Aspartate metabolism produces essential components for pepdidoglycan biosynthesis, such as diaminopimelic acid, and components of the aspartate pathway represent possible drug targets [66].

In summary, the above results suggest that ranalexin exposure induced a cell wall stress response, similar to that associated with cell wall active antibiotics or glycopeptide intermediate-resistant *S. aureus *(GISA) strains. Follow-up laboratory experiments were performed to further investigate these findings (below).

### Further investigation of ranalexin cell wall effects

**Figure 4:**
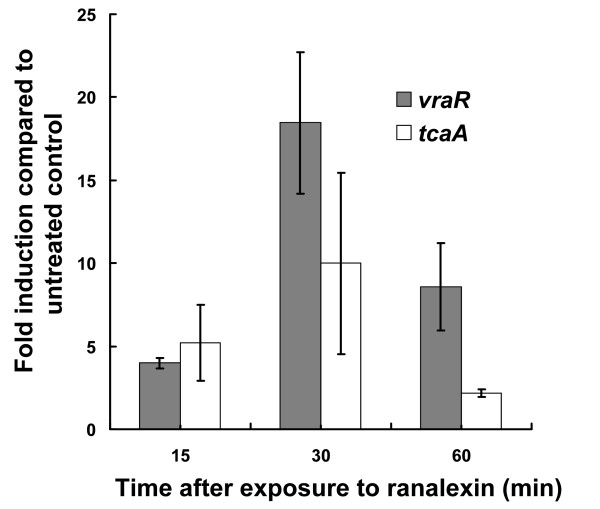
**Quantitative RT-PCR confirms upregulation of *vraR *and *tcaA *by ranalexin**. Fold induction of *vraR *and *tcaA *expression measured by qRT-PCR after 15, 30 and 60 min exposure to 20 μg ml^-1 ^ranalexin. Gene expression was normalised against 16SrRNA and fold-induction upon exposure to ranalexin treatment was then determined relative to untreated controls. The experiment was performed in triplicate and the data shown represents the mean and standard error of the mean.

The physiological relevance of ranalexin induced cell wall related gene and protein expression changes was investigated by producing *vraR *and *tcaA *disruption mutants and subsequent examination for peptide sensitivity. Prior to making mutants, ranalexin induced enhanced expression was verified by quantitative PCR for *vraR *(previously detected by iTRAQ) and *tcaA *(detected by microarray analysis) (Figure 4). In both cases, we again detected ranalexin dependent induction of expression after 15 minute (min) exposure that peaked after 30 min and subsequently declined after 60 min.

The Sigma TargeTron system was applied to generate disruption mutants, for which the *S. aureus *laboratory strain RN4220 was recommended by the manufacturer. Thus, the corresponding genes from MRSA-252 were identified in RN4220 and disrupted. *VraR *disruption induced sensitivity to both ranalexin (Additional file 2 Figure S3) and vancomycin (data not shown), compared to the parent strain. Complementation experiments were attempted, but were inconclusive due to phenotypic effects induced by the empty cloning vector. Growth of the *vraR *mutant was identical to the parent strain in the absence of ranalexin, but was completely inhibited when ranalexin was added (Additional file 2 Figure S3). Similarly, dose response experiments revealed dramatic viability loss for the *vraR *mutant both to increasing concentrations (up to 160 μg ml-1) and to increased duration of exposure (up to 2 h) - contrasting with the parent strain which retained viability (Additional file 2 Figure S3). Considering that the VraRS two-component system regulates a response to cell wall damage [67], these results support the view that ranalexin induces cell wall stress. *TcaA *disruption resulted in minor sensitivity to ranalexin (Additional file 2 Figure S3). No significant differences in rate of loss of viability between the *tcaA *mutant and the parent strain exposed to ranalexin were detected (data not shown). *TcaA *is a member of the cell wall stress stimulon known to be induced by exposure to antibiotics in a VraRS dependent fashion [68]. Therefore, these results suggest that VraRS may be a staphylococcal two-component response regulator involved in cationic peptide resistance. In *S. aureus*, VraRS regulates the transcriptional induction of genes involved in cell wall peptidoglycan biosynthesis and is crucial for resistance to cell wall active antibiotics such as the glycopeptides vancomycin and teicoplanin [55,59,67]. Therefore, these results support the view that ranalexin's mode action includes disruption of the staphylococcal cell wall.

**Figure 5:**
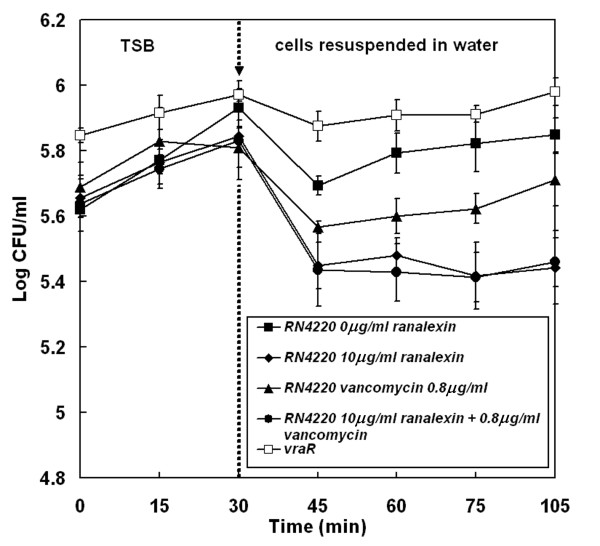
**Ranalexin exposure induces sensitivity to hypo-osmotic stress**. Cells of *S. aureus *RN4220 were exposed to sub-lethal concentrations of ranalexin (10 μg ml^-1^; ♦); vancomycin (0.8 μg ml^-1^; ▲); ranalexin + vancomycin (10 μg ml^-1 ^+ 0.8 μg ml^-1^; ●) in TSB, compared with untreated cells (■) and untreated cells of the *vraR *mutant in TSB (□). After 30 min incubation in TSB cells were harvested, washed twice with distilled water to remove the antimicrobials and resuspended in distilled water. Viable counts were determined throughout the experiment. Error bars represent the standard error of the mean (n = 3).

To further examine ranalexin cell wall effects, osmotic fragility was measured for MRSA cells exposed to sub-lethal concentrations of antimicrobials (Figure 5). Osmotic fragility is a hallmark of cell wall disruption [69,70]. Cultures were incubated in TSB, in the presence and absence of vancomycin or ranalexin, harvested and washed twice in sterile distilled water, followed by incubation in water for 105 min. Viability was determined throughout the experiment. Control cells (RN4220) and *vraR*-disrupted cells showed negligible loss of viability or sensitivity to hypo-osmotic stress. As expected, cells pre-treated with a sub-lethal concentration of vancomycin were sensitive. Notably, pre-treatment with a sub-lethal concentration of ranalexin also induced sensitivity to hypo-osmotic stress (Figure 5). Furthermore, exposure to both ranalexin and vancomycin induced a similar degree of osmotic fragility compared to treatment with ranalexin; suggesting commonalities in modes of action. Coupled with the induction of increased cell wall related gene/protein expression and hypersensitivity produced by *vraR *disruption, these results imply that the inhibitory action of ranalexin is not solely due to membrane disruption [13], but also involves significant effects at the staphylococcal cell wall.

### MRSA persister formation and drug tolerance

Exposure to ranalexin was found to produce strong upregulation of proteins encoded by the *pstSCAB-phoU *operon PstS (SAR1402, 22.10-fold), PstC (SAR1401, 11.73-fold), PstA (SAR1400, 6.47-fold), PhoU (SAR1398, 5.99-fold) and PstB (SAR1399, 8.01-fold) (Additional file 1 Table S1). Four of these (SAR1398, SAR1400-SAR1402) formed a significant network module (*p *≤ 1.2E^-5^, 4/4 nodes RanaUp, Table 1). SAR1399 did not fall into a module but had connections to all the above genes.

PhoU acts as a persister switch in *E. coli *multidrug tolerance and impacts on widespread processes beyond inorganic phosphate (Pi) transport [71]. Genetic disruption of the *pstC *transmembrane component of the Pi transporter with the Sigma TargeTron system did not have any effect on ranalexin sensitivity (data not shown). Combined with the proteome profiling and network analysis this data suggests that MRSA adopts a PhoU-mediated persister phenotype to acquire antimicrobial tolerance, and that upregulation of the Pi transporter is not a major component of this response. Indeed, the *pstSCAB-phoU *operon was induced by *Streptococcus pneumoniae *exposure to penicillin [72], and targeted disruption correlated with increased sensitivity to ciprofloxacin in *Mycobacterium smegmatis *[73]. A pyrimidine metabolism module was RanaDown (*p *≤ 0.028, 3/4 nodes RanaDown) reflecting growth inhibition that is a hallmark of persister formation [74]. Additionally, several other cell division proteins were RanaDown, including SAR0017 (PurA, 0.62-fold, intermodular hub), SAR1047 (PurH, 0.27-fold), SAR2000 (PurB, 0.7-fold), SAR1180 (PyrF 0.82-fold), and the transcript SAR1040 (*purC*, 0.48-fold) was RanaDown. These genes (or operons as applicable) are therefore tentative candidates for negative regulation by PhoU in MRSA-252. The results presented in earlier sections, finding ranalexin exposure upregulated cell wall anabolism and reduced virulence, also align with persister formation. Indeed, persister bacteria exhibit thickening of the cell wall and loss of virulence factors [75]. These findings underline the importance of persister formation in MRSA drug tolerance.

### Multiple actions in MRSA killing

Ranalexin is canonically associated with cell membrane permeabilisation [13]. In agreement with this, a cation antiport module was significant upregulated (*p *≤ 9.0E^-5^, 8/17 nodes RanaUp). The RanaUp genes in this module were subunits A-F (SAR0630-SAR0635) of the Na^+^/H^+ ^antiporter encoded by the *mnh2 *operon, SAR0629 (phage integrase family protein) and subunit G (SAR0908) of the Na^+^/H^+ ^antiporter encoded by the *mnh *operon [76]. The module also included subunits A-F from the *mnh *operon, and three hypothetical proteins (SAR0628, SAR0627, SAR0915). The upregulation of these 'electrogenic monovalent cation proton antiporter-3' operons [77] implies that membrane permeabilisation leading to cation influx and possible dissipation of transmembane electrochemical gradient is a major effect of ranalexin exposure. The *mnh *and *mnh2 *operons are important for Na^+^-dependent pH homeostasis in *B. subtilis *and are induced by alternative sigma factor B (σB) in *S. aureus *[78]. Further, the GO term 'ion transport' (GO:0006811, *p *≤ 0.020, n = 9) was significantly enriched with upregulated genes. Additional to the above genes, these included SAR0723 (*cadA*, 2.06-fold), encoding a probable cadmium-effluxing P-type ATPase that confers resistance to Cd^2+ ^toxicity [79]; SAR0110 (2.14-fold) encoding a putative Na^+^/Pi-cotransporter; SAR0139 (2.09-fold), encoding a putative tetracycline, K^+ ^or Na^+^/H^+ ^antiporter [80]; and SAR2233 (*czrA*, 2.49-fold), encoding a regulatory transcription factor induced in response to Zn^2+ ^stress [81]. A significant module (*p *≤ 0.046, 3/6 nodes RanaUp) contained the *opuC *osmoprotectant transport operon (SAR2535-SAR2538); osmoprotectant transporters are thought to be strongly dependent on σB induction [78,82]. Upregulation of osmoprotectant transport is likely a product of σB induction by cation influx, especially given the observed upregulation of the *mnh *and *mnh2 *operons. Interestingly, increased osmolarity would be expected to act in concert with the ranalexin cell wall effects presented above in producing hypo-osmotic fragility and disruption of cell integrity. Therefore the membrane and cell wall actions of ranalexin may exert complementary effects in killing *S. aureus *MRSA-252.

The response signature included upregulation of the *dlt *operon D-alanine and D-alanyl lipoteichoic acid synthesis, which leads to increased positive charge at the cell surface and reduced peptide binding [83]. Consistent with the expected ranalexin induced stress, a significant module (*p *≤ 0.027, 3/3 nodes RanaUp) contained chaperones and stress response genes. Also, the chaperone proteins GroEL (encoded by SAR2116, 1.56-fold) and FtsH (encoded by SAR0512, 1.23-fold) were RanaUp and intermodular hubs [36]. Another significant module (*p *≤ 9.0E^-5^, 6/7 nodes RanaUp) represented genes for Val/Leu/Ile synthesis (Table 1), which utilizes pyruvate [84], and therefore may allow for greater glycolytic flux in powering ranalexin stressed cells. Indeed, several glucose catabolism and uptake proteins were upregulated, including EIIA-Glc (encoded by SAR1435, 2.65-fold), PGK (encoded by SAR0829, 1.13-fold) an intermodular hub, and PGI (encoded by SAR0924, 1.22-fold) substantiating upregulation of glycolysis upon ranalexin exposure. Interestingly, L-LDH1 (encoded by SAR0234, 0.77-fold) was RanaDown; in conjunction with upregulation of Val/Leu/Ile synthesis and glycolysis this suggests repurposing of pyruvate into production of hydrophobic proteins for peptide sequestration [85]. These results, and those presented in above sections, detail several lines of defence employed by MRSA to escape the effects of cationic AMPs.

In summary, evidence supports multiple inhibitory actions for ranalexin including cell wall effects, increased cation influx and possible dissipation of transmembrane electrochemical gradient. MRSA attempts several survival strategies when faced with antimicrobial peptide (ranalexin) stress, and evidence was found for complementary inhibitory actions in killing MRSA.

## Conclusions

A global gene functional association network that covers 95% of *S. aureus *MRSA-252 genes was presented. Careful benchmarking against blind test datasets found a false positive rate no greater than 3% in the final network. Functional network analysis of drug response signatures enabled novel insights into the mode of action of a cationic antimicrobial peptide (AMP), ranalexin, as well as insights into MRSA-252 antimicrobial resistance mechanisms. Evidence was presented for novel ranalexin effects on the bacterial cell wall, in addition to the previously characterised action at the cell membrane [13], and these actions were proposed to act in concert. These findings agree with previous observations that cationic AMPs exert complex inhibitory actions [86]. The FtsH membrane chaperone was an intermodular hub [36], upregulated in response to ranalexin and suggested as a promising candidate drug target, particularly for combination therapy with cell wall active agents. Indeed, combination therapy has clear advantages for treatment of resistant bacteria [87,88]. Additionally, the two-component response regulator VraR was suggested to be important for mediating tolerance to antimicrobial peptides. Therefore, VraRS may be a staphylococcal two-component response regulator involved in cationic peptide resistance, in addition to apsRS [89,90]. Ranalexin exposure produced MRSA-252 virulence reduction, and twenty-two novel virulence factors were inferred. These novel roles in virulence are supported by several additional lines of evidence, including predicted cellular localisation [38], domain matching [40] and operon structure [41,42]. Finally, we present evidence to support PhoU-mediated persister switching as a mechanism of drug tolerance in MRSA; which merits further investigation as a route towards novel therapeutic targets. This work is informative to the development of therapeutic strategies against *S. aureus*, and demonstrates an elegant approach to study drug resistance and mode of action.

## Methods

### Peptide

Ranalexin was synthesised according to the published sequence [13] by Peptide Protein Research Ltd, Wickham, UK, to >95% purity and verified by HPLC and mass spectrometry. A stock solution of 50 mg ml^-1 ^in water was used.

### Cell growth

*Staphylococcus aureus *MRSA-252 was a gift from Prof. Mark Enright, Imperial College London. A glycerol stock of *S. aureus *MRSA-252 stored at -80°C was streaked and maintained on Tryptone Soya Agar (TSA; Oxoid, Basingstoke, UK).

Starter cultures of *S. aureus *MRSA-252 were grown overnight in 25 ml of Tryptic Soya Broth (TSB; Oxoid, Basingstoke, UK) at 37°C in an orbital shaker at 220 rpm. For iTRAQ protein extraction; four flasks containing 25 ml of fresh TSB were then inoculated with these cells to give an identical starting optical density at 600 nm (OD_600_) of 0.01 in each flask. The cultures were then shaken at 220 rpm until an OD_600 _of 0.3 was reached. At this point 20 μg ml^-1 ^ranalexin was added to two culture flasks and an equivalent volume of water to the remaining two cultures. The cultures were then incubated at 37°C with shaking at 220 rpm. Cultures were harvested when they reached an OD_600 _of 0.55-0.6, for the ranalexin treated cultures this corresponded to 1 hour exposure. Cell viability was measured simultaneously via serial dilution and plating on TSA. Growth of *S. aureus *RN4220 (a gift from Prof. Simon Foster, University of Sheffield), and gene disrupted strains made in this background, were grown to mid-exponential phase in 10 ml TSB and diluted to a starting OD_600 _of 0.001 in 100 μl TSB in a 96-well plate (Greiner). Ranalexin was added and the cell growth monitored and recorded over 24 h at 37°C with constant shaking in a PowerwaveXS (BioTek) plate reader. Data were collected using KC4 v.3.2 software and analysed in Microsoft Excel.

Similarly, viability of *S. aureus *RN4220 and gene disrupted strains with or without ranalexin exposure was measured by serial dilution and plating on TSA.

### Protein expression analysis using isobaric Tags for Relative and Absolute Quantitation (iTRAQ)

Protein extracts were prepared from duplicate, mid-exponential cultures of *S. aureus *MRSA-252 grown in TSB at 37°C (Oxoid) as above. Cells were harvested by centrifugation at 2500 g for 15 min. The cell pellets were washed twice in sterile distilled H_2_O, and proteins extracted by bead beating (Biospec) in triethyl ammonium bicarbonate (TEAB; Sigma) buffer (0.5 M, pH8.5) containing 0.1% w/v SDS. The cells were then pelleted and resuspended in 1 ml fresh buffer. Crude cell extracts were prepared from this cell suspension by bead beating. This procedure was performed using 1.5 ml of glass beads (Biospec) with 4 bursts of 1 min each and 1 min rest intervals on ice. Protein concentrations were determined using the Calbiochem non-interfering protein assay kit and with BSA as standards. Protein was then labelled with iTRAQ reagents following the manufacturer's protocol (Applied Biosystems). Briefly, 20 μl (55 μg) of each sample was reduced and the cysteine residues blocked with MMTS before digesting each sample with trypsin. Following an overnight (16 h) tryptic digestion each sample was labelled with one of 4 isobaric iTRAQ reagents, designated 114, 115, 116 and 117, since each carries a reporter group with approximately these molecular masses. In this experiment, iTRAQ reagents 114 and 115 were used to label the duplicate control samples while 116 and 117 were used to label the duplicate ranalexin treated samples (see Figure 1). After labelling, the 4 samples were combined into one tube and then fractionated by cation exchange chromatography in order to simplify the samples prior to analysis by LC-MS/MS. The combined sample was diluted with 7 ml of 10 mM KH_2_PO_4_, 25% v/v acetonitrile (pH 3.0; adjusted with 1 M H_3_PO_4_) and then loaded onto an equilibrated cation exchange column. The column was then washed with 2 ml of 10 mM KH_2_PO_4_, 25% v/v acetonitrile buffer. Then the labelled peptides were eluted from the column by washing with 12 aliquots of 600 μl elution buffer (1 M KH_2_PO_4_, K_2_HPO_4_), which contained KCl at concentrations of 40, 50, 60, 70, 80, 90, 100, 120, 140, 165, 220 and 280 mM, respectively. These 12 eluted fractions were collected separately and stored at -20°C prior to analysis by LC-MS/MS.

The buffer was removed under reduced pressure and the samples resuspended in 0.5% formic acid for mass spectrometric analysis. Peptides were separated using an UltiMate nanoLC (LC Packings, Amsterdam) equipped with a PepMap C18 trap & column, using a 3.5 hr gradient of increasing acetonitrile concentration, containing 0.1% formic acid (5-35% acetonitrile in 3 hr, 35-50% in a further 30 min, followed by 95% acetonitrile to clean the column). The eluent was sprayed into a Q-Star Pulsar XL tandem mass spectrometer (Applied Biosystems, Foster City, CA) and analysed in Information Dependent Acquisition (IDA) mode.

### Protein identification and quantification

The raw iTRAQ data from Analyst 1.1 (Applied Biosystems) was analysed with ProQuant (Applied Biosystems), this gave confidence scores for peptide identifications and quantitative data based on the 114, 115, 116 and 117 peak intensities found in the MS/MS spectra. The output data were viewed through the ProGroup Report viewer software (Applied Biosystems). The data in the ProGroup report included a list of all proteins identified (with a confidence of 99% or greater) and also the ratios of the level of each identified protein in the control samples (labelled 114 and 115) versus the ranalexin treated samples (labelled 116 and 117). Each ratio was calculated from the ratios of the individual peptides identified as being derived from that protein. In the ProGroup Report all ratios were returned with calculated p-values, which indicated the likelihood that the ratios do not differ significantly from 1. For each protein a total of 4 expression ratios and their corresponding p-values were considered in the analysis; 116/114 (ranalexin_1_/control_1_), 116/115 (ranalexin_1_/control_2_), 117/114 (ranalexin_2_/control_1_), and (117/115 ranalexin_2_/control_2_). These p-values were combined by Fishers method and proteins with Benjamini-Yekutieli false discovery rate corrected p-value > 0.05 were excluded [91]. A further filtering step excluded proteins where any of the four ratios described above had p-value > 0.05. Protein expression changes were expressed as the mean of the four statistically significant expression ratios. iTRAQ data are available from the PRIDE database (accession numbers 14807-14816) [92].

### RNA extraction for microarrays

For RNA extraction (in triplicate); 10 ml of TSB was inoculated to an OD_600 _of 0.01, and, similar to the iTRAQ experiment above, 20 μg ml^-1 ^ranalexin was added at an OD_600 _of 0.3 and cells harvested after 30 min incubation with peptide. 20 mL RNAprotect (Qiagen) was added to the 10 ml cultures and processed according to manufacturer's instructions. Pellets were stored frozen at -80°C. Pellets were thawed at room temperature and washed in TE buffer (1 ml 10 mM Tris, 1 mM EDTA, pH 8.0). After removing the supernatant, pellets were resuspended in TE containing 200 μg ml^-1 ^lysostaphin, 400 units ml^-1 ^mutanolysin and 40 μg ml^-1 ^proteinase K and incubated at 37°C for 90 min with gentle mixing. Then 4 ml RLT buffer from the RNeasy midi kit was added to the mixture and processed according to manufacturer's instructions. This was followed by on-column DNA digestion using RNase-free DNase (Qiagen), according to manufacturer's instructions. RNA was eluted in 250 μl RNase-free water and eluted again using the same 250 μl. The concentration and the quality of the RNA was measured using an Agilent 2100 Bioanalyzer (Agilent Technologies).

### Microarray hybridisation and analysis

cDNA was synthesised from the RNA extracted from both control and ranalexin treated cultures using SuperScript II and random hexamers (Invitrogen) and labelled with either Cy3 or Cy5-dCTP (GE Healthcare). Three independent biological replicates of the control culture and ranalexin treatment were performed and the RNA derived from these was labelled and co-hybridised as two dye-swapped technical replicates, providing a total of six arrays for analysis. Hybridisation, washing and scanning of the *S. aureus *microarray (SAv1.1.0) was undertaken as described previously [93]. The array design is available in BμG@Sbase (accession number: A-BUGS-17; http://bugs.sgul.ac.uk/A-BUGS-17) and also ArrayExpress (accession number: A-BUGS-17).

Feature extraction was performed using ImaGene v5.5 (BioDiscovery) and the microarray data were normalised and statistically analysed using GeneSpring v7 software (Agilent Technologies). Data points that were flagged by ImaGene as present or marginal were only included in the analysis. The data were median normalised and the averaged normalised expression ratios of ranalexin versus control were calculated for each gene. To identify differentially expressed genes in response to ranalexin treatment, the data were filtered to detect genes that had a greater than two-fold expression difference with a t-test p-value < 0.05 when the Benjamini & Hochberg false discovery rate correction [94] was applied. Fully annotated microarray data has been deposited in BμG@Sbase (accession number: E-BUGS-65; http://bugs.sgul.ac.uk/E-BUGS-65) and also ArrayExpress (accession number: E-BUGS-65).

### MRSA-252 gene functional association network

A graph was constructed by a supervised learning approach in order to model MRSA-252 global gene function. Genes were nodes and edges represented functional association in signalling and metabolic pathways. The 2639 MRSA-252 genes in the Uniprot [30] database (taken on 21/11/08) have a possible 3480841 unique pairs; by reference to the UniProt XML we were able to assign co-annotated Gene Ontology (GO) terms for 1358130 pairs (1742 genes). The 679 coannotated GO terms were placed into ten bins each with similar numbers of pairs, and an eleventh bin contained the pairs with no coannotated GO term. Frequency values for each bin were assigned as a fraction of the total pairs. The STRING database (v8.0) [31] had 81290 unique pairwise scores for MRSA-252 (2618 genes). These pairs were placed into ten bins, with an eleventh bin for pairs without STRING scores, and bin frequency values calculated as described above.

Gold-standard datasets to define functionally related and functionally unrelated sets of genes were derived from the 102 MRSA pathways described in KEGG version 48 [32]. Manual inspection of the 102 pathways removed three considered to be overly broad functional groupings. The three excluded pathways were 'ABC transporters' (sar02010), 'Aminoacyl tRNA biosynthesis' (sar00970) and 'Two-component system' (sar02020), additionally ribosomal RNAs were excluded from the pathway 'Ribosome' (sar03010). The positive data, representing functionally related genes, were all remaining pairs within each of the 99 selected MRSA-252 KEGG pathways (9835 unique pairs, 1162 genes). The negative data, representing functionally unrelated genes, were all pairs formed between genes from different pathways (198500 unique pairs, 1162 genes). Uniprot identifiers were mapped to KEGG identifiers by reference to the Uniprot XML [30]. Ten percent from each of the positive and negative datasets was randomly selected from each STRING and GO bin combination (TEST-N). TEST-N was set aside as blind test data not used in the network development. The data remaining after subtraction of TEST-N comprised 8861 positive and 178640 negative pairs (TRAIN-N).

From TRAIN-N, the probabilities that a given pair of genes is functionally associated were estimated from the coannotated GO term frequency bin values (pGO) according to Bayes rule (Equation 1).(1)

Where:

*P(Freq | Int) *is the probability of obtaining the bin frequency value given the genes are functionally related.

*P(Int) *is the prior probability that genes are functionally related.

*P(Freq) *is the prior probability of the bin frequency value.

The same formula was applied to estimate the probability that a given pair of genes is functionally associated from the STRING frequency values (pSTRING). Logistic regression was applied to combine pSTRING and pGO values into a single functional association probability estimate. For this purpose balanced training (TRAIN-B) and testing (TEST-B) datasets were respectively developed by random selection of negative examples from TRAIN-N and TEST-N. The logistic regression model was fitted over TRAIN-B with the R function glm [95] and is given in Equation 2.(2)

Where:

*pGO *is the Bayesian probability that the pair of genes are functionally related from the coannotated GO term data.

*pSTRING *is the Bayesian probability that the pair of genes are functionally related from the STRING data.

Functional association probabilities were generated for all 3480841 unique MRSA-252 gene pairs according to Equation 2, thereby generating the initial network. Additional file 2, Figure S4 shows a receiver-operator characteristic plot for these functional association probabilities over the TEST-N and TEST-B datasets. The F-measure [96] of information retrieval over TRAIN-N was used as a guide to determine a threshold (P ≥ 0.75) to generate the high-confidence network (Additional file 2 Figure S5). This network had 2494 nodes and 19076 edges; the true negative rates over TEST-N, TEST-B were 0.970, 0.983 respectively. A final blind test dataset (TEST-Z, 3403 pairs) included the one completely new MRSA-252 pathway (sar03018) added to KEGG after the network was generated, plus the amino-acyl-tRNA biosynthesis (sar00970) and two-component system (sar02020) pathways which had previously been excluded from the gold-standard (above). The thresholded network true negative rate over TEST-Z was 0.980. The network is available in several formats from Additional File 3 MRSA-252_network.zip.

### Mapping proteomics and microarray data into the functional association network

Of the 103 downregulated MRSA-252 genes from the microarray experiment, 88 shared at least one edge with another network node. Of the 93 upregulated genes, 83 shared at least one edge with another network node.

The 56 upregulated and 15 downregulated proteins from the ProQuant (Applied BioSystems) analysis included identifiers from a mixture of different databases and different strains. These 71 identifiers were mapped to the protein products of 71 unique MRSA-252 genes using stringent sequence searching critera (BLASTP [97]) alignment, 95% query coverage, 98% identity). At least one edge with another network node was shared by 54/56 upregulated and 15/15 downregulated proteins.

### Network modules, significance assessment and intermodular hubs

The MCL algorithm [35] (inflation value (I) of 3, otherwise default parameters) was applied to define 597 putative functional modules, formed from 2005 genes in the high-confidence network. The clustering with I = 3 was chosen because the distribution of module sizes was considered to give a more meaningful representation of MRSA metabolic and signalling pathways. This is because the I = 3 clustering produced comparatively few very large modules. The clustering efficiency reported by the clm program [35] at I = 2 (default value) and I = 3 was very close (0.44, 0.46 respectively). The distribution of module sizes is shown in Additional file 2 Figure S6. Therefore, 489 (19.6%) genes in the network were not assigned to a module. The set of genes significantly affected by ranalexin exposure, as defined by the microarray and proteomics data, were assigned to network modules. The significance of the enrichment of ranalexin affected genes in network modules was estimated empirically by resampling. The background distributions for the microarray data were drawn from the 2012 genes that were both represented in the network and detected on all 6 microarrays. The background distributions for the proteomics data were drawn from the 522 proteins that were both identified at 95% confidence in the iTRAQ experiment and mapped to genes in the network. A total of 100,000 draws of size N were randomly taken from the network, where N is the size of the test gene set. For example, N = 83 for the background distribution generated for the 83 genes in the upregulated microarray set. Four background distributions were thus generated, (corresponding to up- and downregulated gene sets from the microarray and proteomics experiments. p-values estimated from these distributions were false discovery rate corrected [94].

In order to estimate significance of putative modules predicted function for Gene Ontology [27] annotations, Fishers test was applied. The false discovery rate corrected [94] hypergeometric p-value reflects the significance of function enrichment for the given network module.

Intermodular hubs were defined as those nodes in the top ten percent of both degree and betweeness centrality, that also fell outside the modules identified by MCL. Betweenness was calculated using NetworkAnalyzer [98].

### Gene Ontology profiling

The microarray and proteomics datasets were analysed by Gene Ontology (GO) [27] profiling using Fisher's exact test. The microarray reference dataset comprised all genes that were flagged as present or marginal by the feature extraction software (GeneSpring, Agilent) for 6/6 microarrays. The proteomics reference dataset comprised all proteins identified above 95% significance by ProQuant (Applied BioSystems) analysis of the iTRAQ data, including those where expression ratios were not significantly affected by ranalexin treatment. The identifers for molecules in the microarray and proteomics datasets were mapped to UniProt [30] with reference to the UniProt XML, and the PIR [99] mapping to UniProt. The UniProt GO mapping for *Staphylococcus aureus *was used to assign GO terms to the reference datasets. The GO XML was parsed to ascertain the ancestor terms from each GO term assigned. Sequence identifiers without assigned GO terms were discarded. Test datasets comprising those molecules from the proteomics or microarray reference data that were identified as upregulated or downregulated were thus defined: PU (proteomics, upregulated) PD (proteomics, downregulated), TU (microarray, upregulated), TD (microarray, downregulated). Background datasets for each of the four test datasets were defined as the remainder of the relevant reference dataset after subtraction of the test data. The Fisher exact test was performed for every GO term associated with each of the test datasets using R [95]. False discovery rate adjusted [94] one-tailed p-values were calculated to assess enrichment of GO terms; terms with adjusted p-values ≤ 0.05 were considered significant.

### Quantitative RT-PCR

RNA was extracted in triplicate from 5 ml MRSA-252 cultures at OD_600 _that were exposed to 20 μg ml^-1 ^ranalexin for 0, 15, 30 and 60 min. Control cells were harvested at an OD_600 _of 0.6. RNA extraction was carried out as described above except the RNeasy Protect Bacteria Mninkit (Qiagen) was used. RNA was eluted in 100 μl of RNase-free water. The concentration and quality of RNA was measured with a NanoDrop spectrophotometer (NanoDropTechnologies, Wilmington, DE) and RNA diluted to a working concentration of 50 ng μl^-1^. Gene specific and control primers were synthesised by Sigma-Aldrich: (*vraR*: Forward: AGATATCGCCGATGCAGTTC, *vraR*: Reverse: CTCTGCGCGCTTTTTCATAC, *tcaA*: Forward: CGGACAACAAGCACAAGATG, *tcaA*: Reverse: CCCAAGGCACCATTTTTCTC, 16SrRNA: Forward: CCAGCAGCCGCGGTAAT, 16SrRNA: Reverse: CGCGCTTTACGCCCAATA).

qRT-PCR was performed with the iScript™ One-Step RT-PCR Kit With SYBR^® ^Green (Bio-Rad). Duplicate PCR reactions, each using 100 ng of RNA from triplicate RNA extractions, were carried out using a Bio-Rad iCycler iQ under the following conditions; cDNA synthesis: 10 min at 50°C; iScript reverse transcriptase inactivation: 5 min at 95°C; PCR cycling and detection (35 cycles): 10 sec at 95°C: 30 sec at 60°C. Melt curve analysis: 1 min at 95°C, 1 min at 55°C, 10 sec at 55°C (80 cycles, increasing each by 0.5°C each cycle). Data was analysed using Bio-RAD iQ5 software.

Ct values of test genes were normalised against 16s rRNA expression using the model described in [100] to calculate fold differences in transcript levels in the ranalexin treated samples over the untreated control. Experiments were performed in triplicate.

### Gene disruption

Gene disruptions were performed using the TargeTron system (http://www.sigmaaldrich.com/sigma-aldrich/areas-of-interest/life-science/functional-genomics-and-rnai/targetron.html; Sigma Aldrich) and the pNL9164 vector (Sigma Aldrich), according to manufacturer's instructions with the following variation: PCR products generated were ligated into pNL9164 using T4 ligase (Promega) and transformed into *E. coli *dH5α (Invitrogen). Propagated plasmids were then transformed into *S. aureus *strain RN4220 using a Micropulser (Bio-Rad). The examined genes were highly conserved between the two strains, while RN4220 strongly expresses the v*raSR *system and tcaA in response to cell wall active agents [54,101]. For example, to disrupt *vraR*, two sets of primers were selected from those designed by the proprietary TargeTron website. One of these sets, where the insertion was between bases 237 and 238 of *vraR *gave positive results in a diagnostic PCR, with the gene-disrupted strain giving a PCR product 900 bp larger than that of the parental strain. Once positive disruptants had been identified the pNL9164 plasmid was cured according to the manufacturer's protocol. An identical procedure was employed to generate disrupted strains for other genes of interest. All gene disruptions were verified by PCR.

## Abbreviations

σB: alternative sigma factor B; AMP: Antimicrobial Peptide; FPR: False Positive Rate; GO: Gene Ontology; iTRAQ: isobaric Tags for Relative and Absolute Quantitation; LC-MS/MS: Liquid Chromatography tandem Mass Spectrometry; MRSA: Methicillin Resistant *Staphylococcus aureus*; Pi: inorganic phosphate; RanaDown: significantly downregulated upon ranalexin exposure; RanaUp: significantly upregulated upon ranalexin exposure; TSB: Tryptic Soya Broth;

## Authors' contributions

PC conceived the overall project and designed the experiments performed by SG, CB and SS. SG performed and helped design the cell culture, qPCR, microarray, hypo-osmotic stress and gene disruption experiments. KG and JH helped perform and design the microarray experiments. CB and SS performed and helped design the iTRAQ experiments. IO conceived, designed and performed the data integration, gene ontology profiling, MRSA gene network, and network-based analyses. IO suggested the hypo-osmotic stress experiment. GB helped design the gene ontology profiling and gene network. IO and PC wrote the first draft of the manuscript. All authors read and approved the final manuscript.

## Supplementary Material

Additional file 1 **Supplementary tables**. Table S1 summarises the Ranalexin dependent changes in MRSA-252 protein expression, Tables S2 and S3 respectively summarise the Ranalexin dependent up and downregulation of gene expression in MRSA-252, Tables S4-S7 summarise the significant GO terms for the above sets of up/downregulated proteins and genes, Table S8 gives the intermodular hubs identified from the MRSA-252 gene functional association network. Click here for file

Additional file 2 **Supplementary figures**. Figure S1 shows the network degree distribution, Figure S2 shows the network clustering coefficient distribution, Figure S3 shows the increased sensitivity of gene disruption mutants to ranalexin. Figure S4 shows Receiver Operator Characteristic Plots for the unthresholded network, with blind test datasets TEST-N (real-world distribution of non-interacting and interacting genes) and TEST-B (balanced distribution). Figure S5 shows the F-measure over the TRAIN-N dataset, which was used to determine the edge threshold for the high-confidence network. Figure S6 shows the network module size distribution. Click here for file

Additional file 3 **MRSA-252 gene functional association network**. A zip archive of the functional association network in tabdelimited, SIF and GML formats. Click here for file
