# Supplementary material for: Global network analysis of drug tolerance, mode of action and virulence in methicillin-resistant S. aureus
Source: BMC Syst Biol. 2011 May 12;5:68. doi: 10.1186/1752-0509-5-68 (PMC3123200; doi:10.1186/1752-0509-5-68)
Supplement: Additional file 2 — Supplementary figures. Figure S1 shows the network degree distribution, Figure S2 shows the network clustering coefficient distribution, Figure S3 shows the increased sensitivity of gene disruption mutants to ranalexin. Figure S4 shows Receiver Operator Characteristic Plots for the unthresholded network, with blind test datasets TEST-N (real-world distribution of non-interacting and interacting genes) and TEST-B (balanced distribution). Figure S5 shows the F-measure over the TRAIN-N dataset, which was used to determine the edge threshold for the high-confidence network. Figure S6 shows the network module size distribution. [file 1752-0509-5-68-S2.PDF]

Supplementary Figures For 'Global Network Analysis of Drug Tolerance, Mode of Action and Virulence in MRSA'

Table of Contents

Supplementary Figures

Figure S1 Network Degree Distribution 2
Figure S2 Network Clustering Coefficient Distribution 3
Figure S3 Disruption of *vraR* and *tcaA* confers ranalexin sensitivity 4
Figure S4 Raw Network Blind Test Datasets 5
Figure S5 F-measure for Edge Threshold Determination 6
Figure S6 Network Module Size Distribution 7

Supplementary Figures

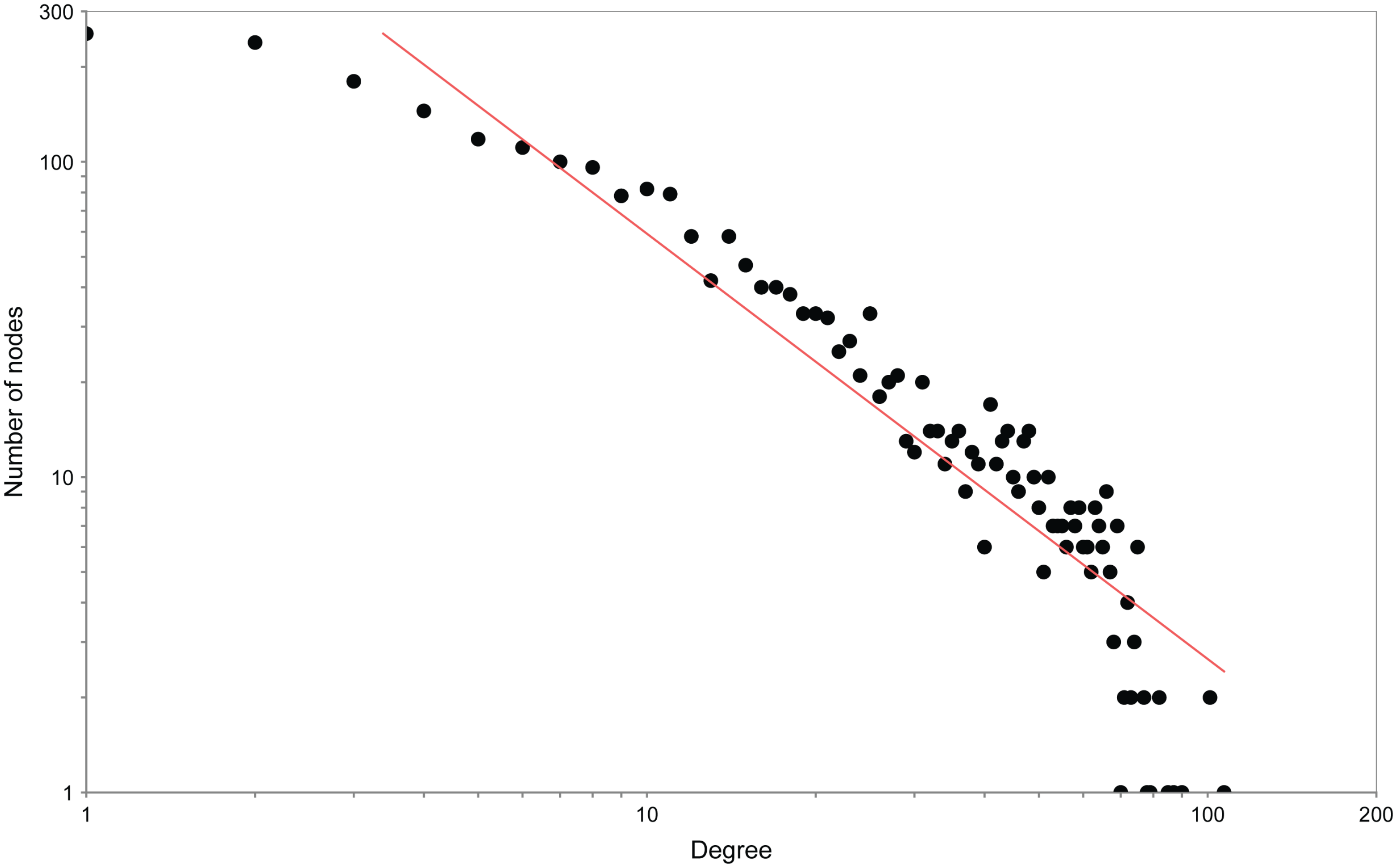

Figure S1. Network Degree Distribution. The red line shows a power law that has been fitted to the network degree distribution (correlation = 0.818). The function y = 1320x-1.35 describes the scaling of the degree, which is a characteristic feature of hierarchical (and scale-free) networks (Yamada & Bork 2009). This figure was generated with NetworkAnalyzer (Assenov et al. 2008), a plugin to Cytoscape (Shannon et al. 2008).

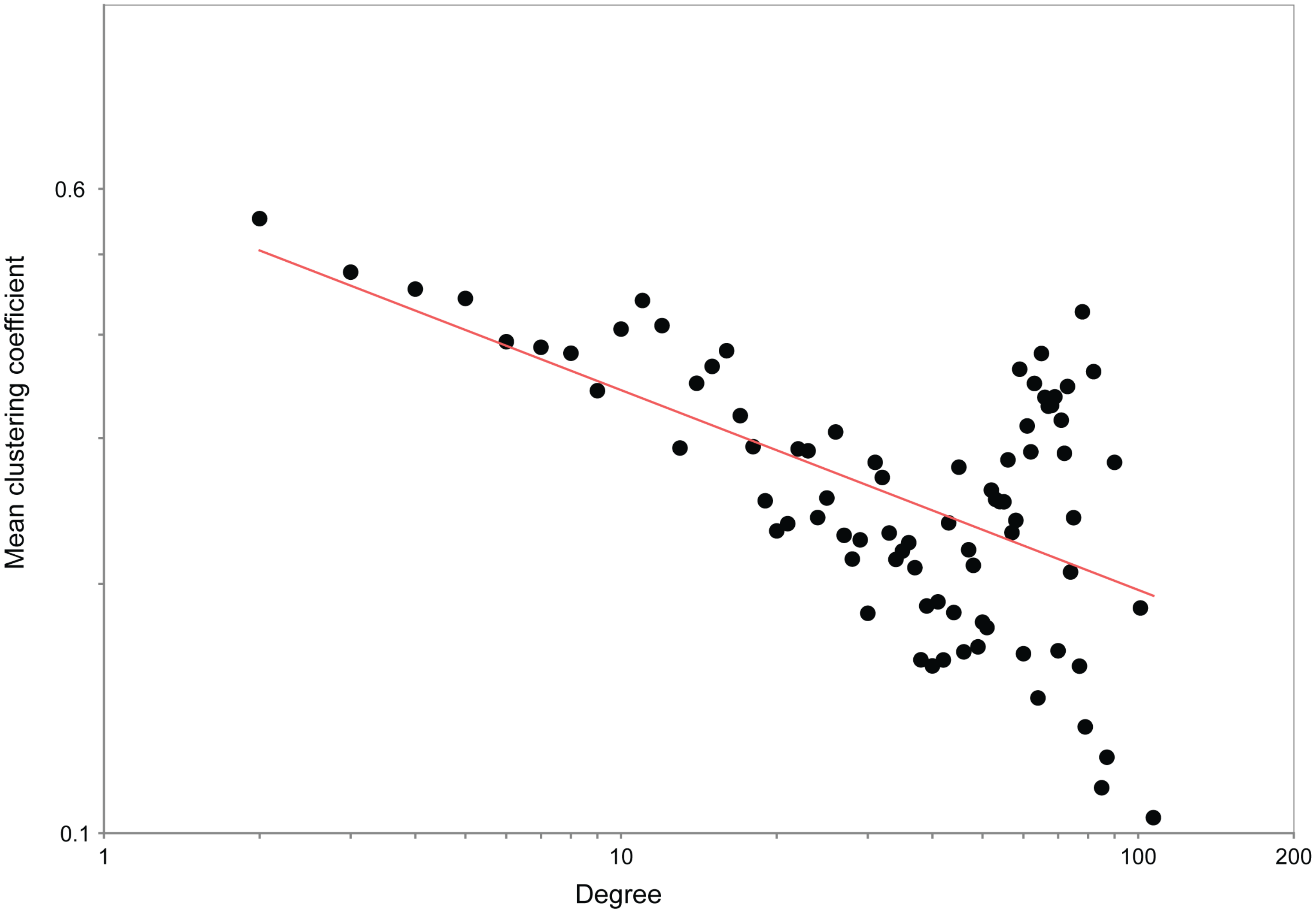

Figure S2. Network Clustering Coefficient Distribution. The red line shows a power law that has been fitted to the network clustering coefficient distribution (correlation = 0.663). The scaling of the clustering coefficient can be described by the function y = 0.6x-0.24, and is consistent with hierarchical modularity (Yamada & Bork 2009). This figure was generated with NetworkAnalyser (Assenov et al. 2008), a plugin to Cytoscape (Shannon et al. 2008).

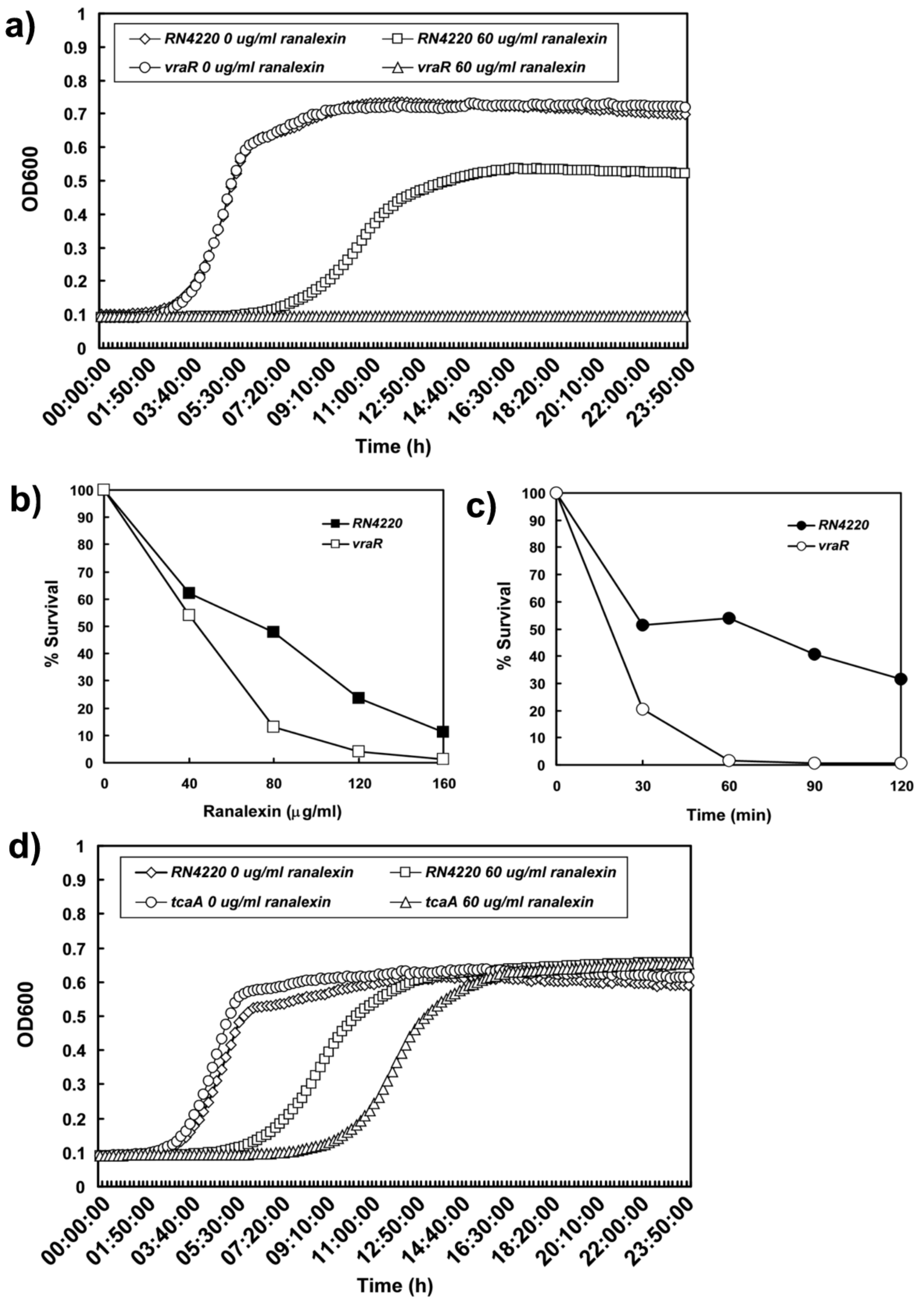

Figure S3. - Disruption of vraR and tcaA confers ranalexin sensitivity.
a) Growth, measured by change in optical density (600nm), of the parent strain RN4220 (◇) and a *vraR* mutant (○) in the absence of ranalexin, or in the presence of 60µg ml^{-1} ranalexin (□, △respectively). Time is indicated in hours: minutes: seconds. A representative result of triplicate experiments is shown. The effect on viability of exposing RN4220 (filled symbols) and the vraR mutant (open symbols) to ranalexin: b) the effect of exposure to increasing ranalexin concentration (0-160µg ml^{-1} in TSB) for 1 h; c) the effect of increasing duration of exposure (0-120 min) to 120µg ml^{-1} ranalexin in TSB. In each case, representative results from duplicate experiments are shown. d) Growth of RN4220 (◇) and a *tcaA* mutant (○); in the absence of ranalexin, or in the presence of 60µg ml^{-1} ranalexin (□, △ respectively). A representative result of triplicate experiments is shown.

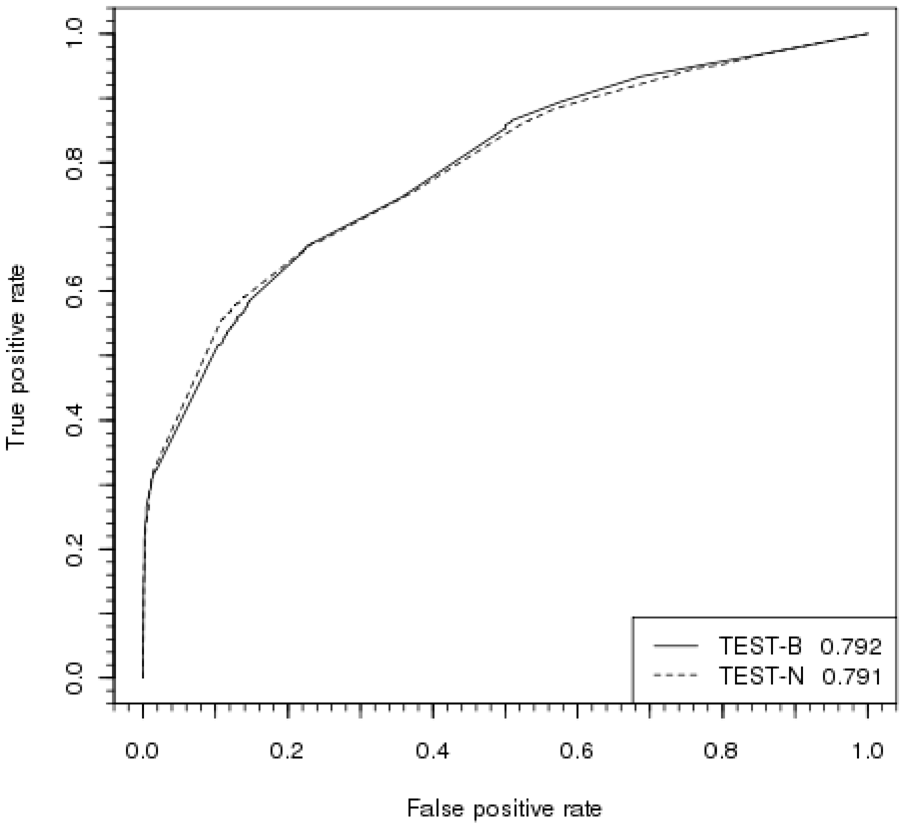

Figure S4. Predictive Power Over Unthresholded Network Receiver Operator Characteristic Plots are shown for blind test datasets TEST-N (real-world distribution of non-interacting and interacting genes) and TEST-B (balanced distribution). Neither TEST-N nor TEST-B was used in any part of the network development process. Areas under the curve are given at the bottom right of the figure (TEST-N 0.791, TEST-B 0.792). This figure was generated in R (R Development Core Team 2010).

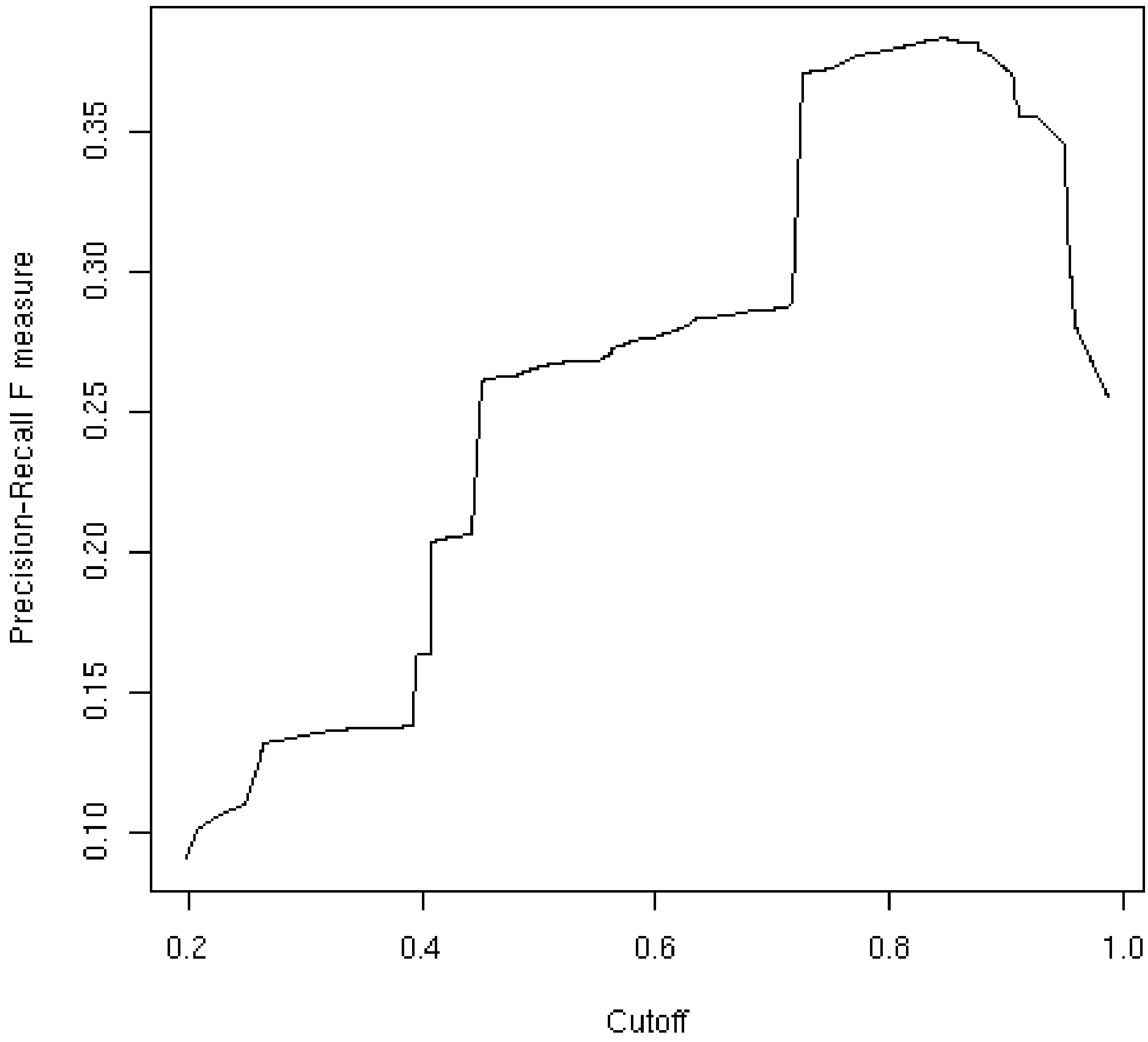

Figure S5. F-measure for Edge Threshold Determination. The F-measure (van Rijsbergen 1979) is shown over TRAIN-N. The edge threshold was determined by reference to the F-measure, FPR and number of edges over TRAIN-N in order to generate the high-confidence network. A threshold value of 0.75 was determined, corresponding to the left side of the 'plateau' with values of F-measure >0.37. This threshold value was chosen to maximise the number of network edges while maintaining high F-measure and low false positive rate. This figure was generated in R (R Development Core Team 2010).

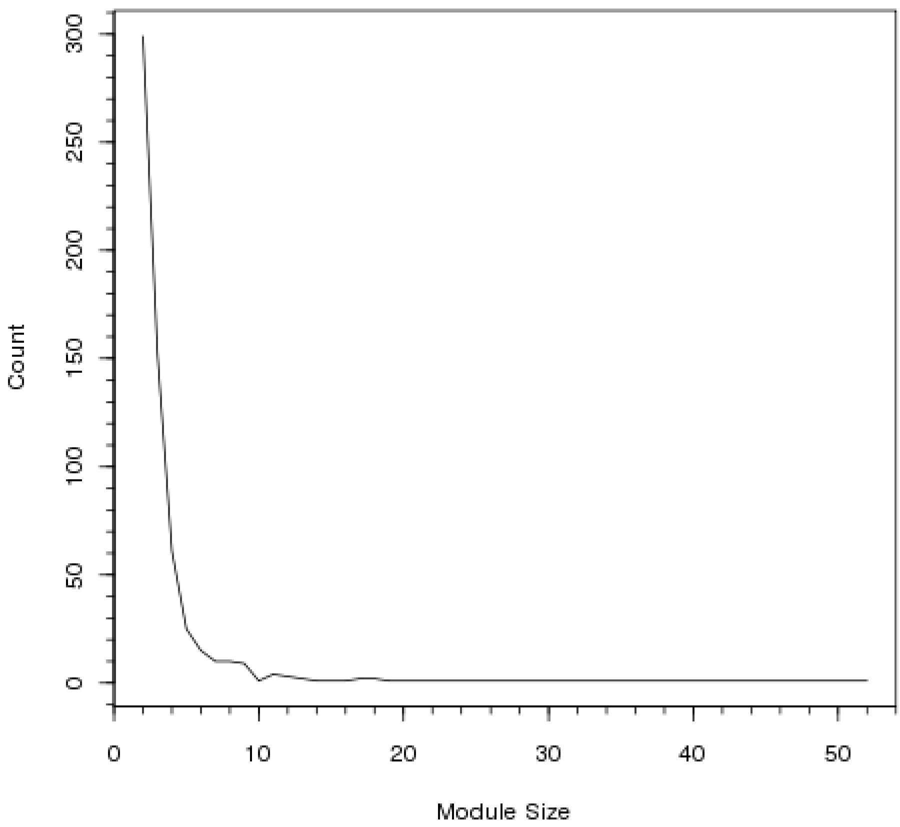

Figure S6. Network Module Size Distribution. The above figure outlines the distribution of 597 clusters (size 2 or more) identified by MCL. One module of size 52 was identified, but there were no modules with size 19-51. This figure was generated in R (R Development Core Team 2010).
